# Necrology

**Published:** 1896-03

**Authors:** 


### NECROLOGY.

At Jersey City, January 30, 1896, W. T. J. McLaughlin, V.S. Dr. McLaughlin was a graduate of the New York College of Veterinary Surgeons, and at the time of his death held a position as Government Meat-Inspector at the Jersey City abattoirs.

The death of Dr. Jno. N. Navin, at Indianapolis, Ind., on January 4th, at the ripe old age of eighty-five years, removes from the veterinary profession one of its oldest members. Dr. Navin always evinced a keen and observant interest in the all-important question of horseshoeing. His pen was always actively wielded in the interest of his adopted vocation, and several books were issued, relative to the diseases of horses, that served a good purpose at the period when they were issued.

Dr. Nat. Carlin, of St. Louis, Mo., died of pneumonia on January 31st. He was well-known among turfmen, and was well-known as a writer of pedigrees. At one time he had general charge of General Grant's stock-farm in Missouri.

